# Association of longitudinal trajectories of fasting plasma glucose with all-cause and cardiovascular mortality among a Chinese older population: a retrospective cohort study

**DOI:** 10.1186/s12889-024-18823-0

**Published:** 2024-05-17

**Authors:** Xuejiao Chen, Jiacheng Ding, Zhan Shi, Kaizhi Bai, Songhe Shi, Qingfeng Tian

**Affiliations:** 1 Department of Epidemiology and Health Statistics, College of Public Health, Zhengzhou University, Zhengzhou, Henan People’s Republic of China; 2 Department of pharmacy, Zhengzhou people’s hospital, Zhengzhou, Henan People’s Republic of China

**Keywords:** Cardiovascular risk, Fasting plasma glucose, Trajectories

## Abstract

The association between fasting plasma glucose (FPG), an important indicator of overall glycemic status, and the risk of cardiovascular mortality has been well investigated. The longitudinal study can repeatedly collect measured results for the variables to be studied and then consider the potential effects of intraindividual changes in measurement. This study aimed to identify long-term FPG trajectories and investigate the association between trajectory groups and cardiovascular and all-cause mortality. A latent class growth mixture modeling (LCGMM) was used to identify FPG trajectories. Cox proportional hazard models were used to estimate associations between FPG trajectories and the risk of all-cause and cardiovascular mortality. A U-shaped relationship between FPG and all-cause and cardiovascular mortality was observed in the restricted cubic spline regression models. Two FPG longitudinal trajectories of low-level (mean FPG = 5.12mmol/L) and high-level (mean FPG = 6.74mmol/L) were identified by LCGMM. After being adjusted for potential confounders, compared with the low-level category, the hazard ratios (HRs) for all-cause and cardiovascular mortality were 1.23(1.16–1.30) and 1.25(1.16–1.35), respectively, for the high-level group. Long-term FPG trajectories are significantly associated with and potentially impact the risk of all-cause and cardiovascular mortality.

**Supplementary Information:**

The online version contains supplementary material available at 10.1186/s12889-024-18823-0.

## Introduction

With a rapidly aging global population and epidemiologic changes in disease, cardiovascular disease (CVD) remains a significant cause of both morbidity and mortality globally, especially for middle-aged and older adults [1, 2], which also causes a substantial economic burden on society [3, 4]. Studies have suggested that FPG is a valuable predictor of CVD in men and women. As a result, a growing number of population-based epidemiological studies are starting to focus on the relationship between fasting glucose and mortality [5–7]. Previous studies have shown that people with high blood glucose are at higher risk of CVD and that there is a J-shaped or U-shaped association between FPG and death [6, 8–11].

However, most are based on a cross-sectional design or assessed at two points baseline and outcome time [11, 12]. The longitudinal study design provides the opportunity to collect measured results for the variables to be studied repeatedly and then take into account the potential effects of intraindividual changes in measurement [13–15]. Group-based trajectory modeling techniques, such as LCGMM, are a universal approach to illustrate the development of the variable over time and can be used to disentangle underlying population heterogeneity [16, 17].

Previous studies have reported that longitudinal trajectories of FPG are associated significantly with incident myocardial infarction, and people with an elevated-level trajectory of FPG are at a higher risk of mortality [7, 18, 19]. In 2018, Lee et al. identified five distinct trajectories of FPG variability and found that compared to the low FPG variability trajectory, the other four trajectories all had significantly higher mortality risks [19]. More recently, Soshiro et al. found that people with sharply increased FPG trajectories were at higher risk for CVD and suggested that studies focus on changes in FPG over multiple time points [7]. However, few relevant longitudinal studies have been conducted, especially among the middle-aged and older Chinese population. Therefore, the primary aim of this study was to identify longitudinal trajectories of FPG and then estimate the associations of FPG trajectories with all-cause and cardiovascular mortality.

## Materials and methods

### Participants

This retrospective cohort study was performed in a dynamic population based on an annual health check-up project, and was carried out since 2010 in Xin zheng, Henan Province. All participants were asked to complete a questionnaire and to take anthropometric and laboratory measurements at baseline and follow-up. Details of this dynamic cohort have been described previously [20–22]. The data were analyzed from residents’ electronic health records in the Xin zheng Hospital Information System from January 2010 to December 2019. To ensure the quality of the cohort and trajectories, the records with missing data for FPG were removed, and each study participant had one health examination record per year. Between January 2010 and December 2019, we followed a total of 101,967 study participants. We excluded 50,488 individuals who met with any one of the following circumstances: the number of medical examinations was 1(*n* = 18,269), 2(*n* = 14,816) and 3(*n* = 12,934); missing information (*n* = 4,469) on body mass index (BMI), waist circumference (WC), smoking, drinking, physical activity, marital status or hypertension at baseline. The number of new participants in the cohort each year and the total number of follow-up visits each year are shown in Table S1. Finally, between January 2010 and December 2019, a total of 51,479 participants with four or more medical records were enrolled.

### Data collection

Data were collected through a standardized questionnaire, as well as from physical and laboratory examinations. Standardized questionnaires of the National Norms for Basic Public Health Services (third edition), which included their sociodemographic characteristics (age, sex), medical history (coronary heart disease (CHD), stroke and hypertension), smoking, drinking, and physical activity, were administered by trained research staff. Based on self-reported marital status, smoking, and drinking, participants were classified as follows: living with a partner or without a partner; nonsmokers (including previous smokers) or current smokers; and never, occasionally, or daily drinkers. The frequency of physical activity was described as never, occasionally, and daily [23].

Standing height and weight were measured to the nearest 0.1 cm and 0.1 kg with the participant standing erect in bare feet, and the results were recorded by the mean of two measurements. BMI was calculated as weight (kg) divided by height squared (m). WC was measured to the nearest 0.1 cm at the midpoint between the lowest rib margin and the iliac crest following a standard protocol. After an overnight fast of 8 h or more, blood samples for the laboratory were obtained to assess levels of FPG using an automatic biochemical analyzer (DIRUI CS380, Changchun, China) [21].

### Assessment of outcomes

The primary outcomes in the study were all-cause and CVD mortality, where CVD death was defined as death from CHD or stroke. For mortality surveillance, participants’ mortality information was obtained from the Xinzheng Center for Disease Control and Prevention from the baseline survey to October 7, 2022. The causes of death were recorded using codes from the International Classification of Diseases (ICD-10), in which death from CVD was defined as I20eI25 and I60eI69.

### Statistical analyses

For non-normal distribution, continuous variables are characterized by the median (interquartile range (IQR)), while categorical variables are expressed as frequency (%). The Kruskal-Wallis test was used to compare continuous variables and the chi-square test for categorical variables.

The latent class growth mixture modeling (LCGMM) was used to explore heterogeneity in the dynamic course of FPG to distinguish subgroups of similar underlying FPG trajectories as experienced over time. Models were fit using the package “lcmm” (version 2.0.0) in R to group participants with a similar trajectory of FPG development from the first examination to the fourth [16]. Three possible polynomial specifications were allowed to describe the longitudinal FPG response as a function of time: a linear, quadratic, and a cubic specification, and every polynomial model (order 1 to 3) was respectively modeled as a 1 to 4 class solution. The choice of the best model was evaluated by the following composite criteria: (1) observing improvement in the Bayesian information criterion (BIC); (2) entropy > 0.7; (3) at least 10% of the participants in each trajectory class; (4) values of mean posterior class membership probabilities; and (5) confirming visually distinct trajectories [24, 25]. For ease of interpretation, we assigned labels to these trajectories based on their modeled graphic patterns, namely low-level and high-level. Cox proportional hazards models were used to estimate HRs and 95% confidence intervals (CIs) between trajectory groups and all-cause and cardiovascular mortality after inspection of Schoenfeld residuals. Model 1 was adjusted for age and gender; Model 2 was adjusted for age, gender, marital status, BMI, smoking, alcohol consumption, physical activity and hypertension. Model 3 was adjusted for age, gender, marital status, BMI, smoking, alcohol consumption, physical activity, hypertension and FPG. To assess nonlinearity, we performed a restricted cubic spline to the multivariable cox proportional hazards models and then the cut-off value was estimated by trying all possible values and choosing the cut-off point with the highest likelihood. Based on bootstrap resampling, cross-validation was applied to assess and compare the discriminative power of model one and model three on the same data set. To investigate potential modification effects of sex on the associations between trajectory groups and all-cause and cardiovascular mortality, we performed subgroup analyses based on sex. To verify the robustness of the results, we conducted an additional sensitivity analysis after excluding those participants with less than four years of follow-up. *P* < 0.05 for a two-sided test was regarded as statistically significant. All analyses were performed using R version 4.1.3 (R Foundation for Statistical Computing).

## Results

The baseline characteristics of the study sample, stratified by all-cause and cardiovascular mortality, are summarized in Table 1. A total of 51,479 study participants (women: 27,792) were included in the present study. The median age (interquartile range) for women and men was 67.6 (61.9–72.0) and 67.4 (62.1–71.5), respectively. During the 322,218 person-years of follow-up (median follow-up time 6.26 years), 6,557 deaths were recorded, of which 3,379 were due to CVD, and 2,384 and 1,161 cases of CHD and stroke, respectively. Compared with participants who survived to the end of the study, the decedents were older, were more likely to be male, lived with a partner, and had a lower BMI. Similar demographic characteristics were observed in participants who died from CVD.

**Table 1:** Baseline characteristics of the study population stratified by outcome

Variables	All-cause mortality		*P* value	Cardiovascular disease mortality		*P* value
	No(*n* = 42,412)	Yes(*n* = 9,067)		No (*n* = 46,667)	Yes (*n* = 4,812)	
Age (years)	64.6 (61.7,69.6)	73.0 (66.2,78.4)	< 0.001	65.1 (61.8, 70.7)	73.0 (66.3, 78.4)	< 0.001
Gender (%)			< 0.001			< 0.001
Women	23,557 (55.5)	4,235 (46.7)		25,460 (54.6)	2332 (48.5)	
Men	18,855 (44.5)	4,832 (53.3)		21,207 (45.4)	2480 (51.5)	
Marital status (%)			< 0.001			< 0.001
Living without partner	9163 (21.6)	3166 (34.9)		10,592 (22.7)	1737 (36.1)	
Living with partner	33,249 (78.4)	5901 (65.1)		36,075 (77.3)	3075 (63.9)	
Smoking (%)			< 0.001			0.047
Never or previous	36,311 (85.6)	7583 (83.6)		39,838 (85.4)	4056 (84.3)	
Current	6101 (14.4)	1484 (16.4)		6829 (14.6)	756 (15.7)	
Drinking (%)			< 0.001			< 0.001
Never	39,403 (92.9)	8371 (92.3)		43,354 (92.9)	4420 (91.9)	
Occasionally	2048 (4.8)	381 (4.2)		2229 (4.8)	200 (4.2)	
Daily	961 (2.2)	315 (3.5)		1084 (2.3)	192 (4.0)	
Physical activity (%)			< 0.001			< 0.001
Never	31,298 (73.8)	7044 (77.7)		34,701 (74.3)	3641 (75.7)	
Occasionally	4237 (10.0)	901 (9.9)		4600 (9.9)	538 (11.2)	
Daily	6877 (16.2)	1122 (12.4)		7366 (15.8)	633 (13.2)	
Hypertension			< 0.001			< 0.001
No	19,717 (46.5)	4432 (48.9)		22,008 (47.2)	2141 (44.5)	
Yes	22,695 (53.5)	4635 (51.1)		24,659 (52.8)	2671 (55.5)	
FPG trajectories			< 0.001			< 0.001
Low-level	32,530 (76.7)	6761 (74.6)		35,755 (76.6)	3536 (73.5)	
High-level	9882 (23.3)	2306 (25.4)		10,912 (23.4)	1276 (26.5)	
FPG (mmol/L)	5.30 (4.8, 5.9)	5.3 (4.8, 6.0)	0.749	5.3 (4.8, 5.9)	5.3 (4.8, 6.0)	0.241
Mean FPG (mmol/L)	5.3 (4.9, 5.9)	5.3 (4.9, 5.9)	0.336	5.3 (4.9, 5.9)	5.3 (4.9, 6.0)	0.213
WC (cm)	83.0 (78.0,90.0)	80.00 (75.0,87.0)	< 0.001	83.0 (77.5,90.0)	80.0 (75.0, 87.0)	< 0.001
BMI (kg/m²)	24.2 (22.4,26.6)	23.4 (21.6,25.8)	< 0.001	24.1 (22.3,26.5)	23.6 (21.8,26.0)	< 0.001
Time of follow-up (years)	6.1 (5.9, 8.0)	5.9 (4.1, 7.0)	< 0.001	6.1 (5.6, 8.0)	6.0 (4.4, 7.0)	< 0.001

Abbreviations: FPG, fasting plasma glucose; BMI, body mass index; WC, waist circumference; Data are presented as median (interquartile range), or number (percentage).

**Fig. 1:**
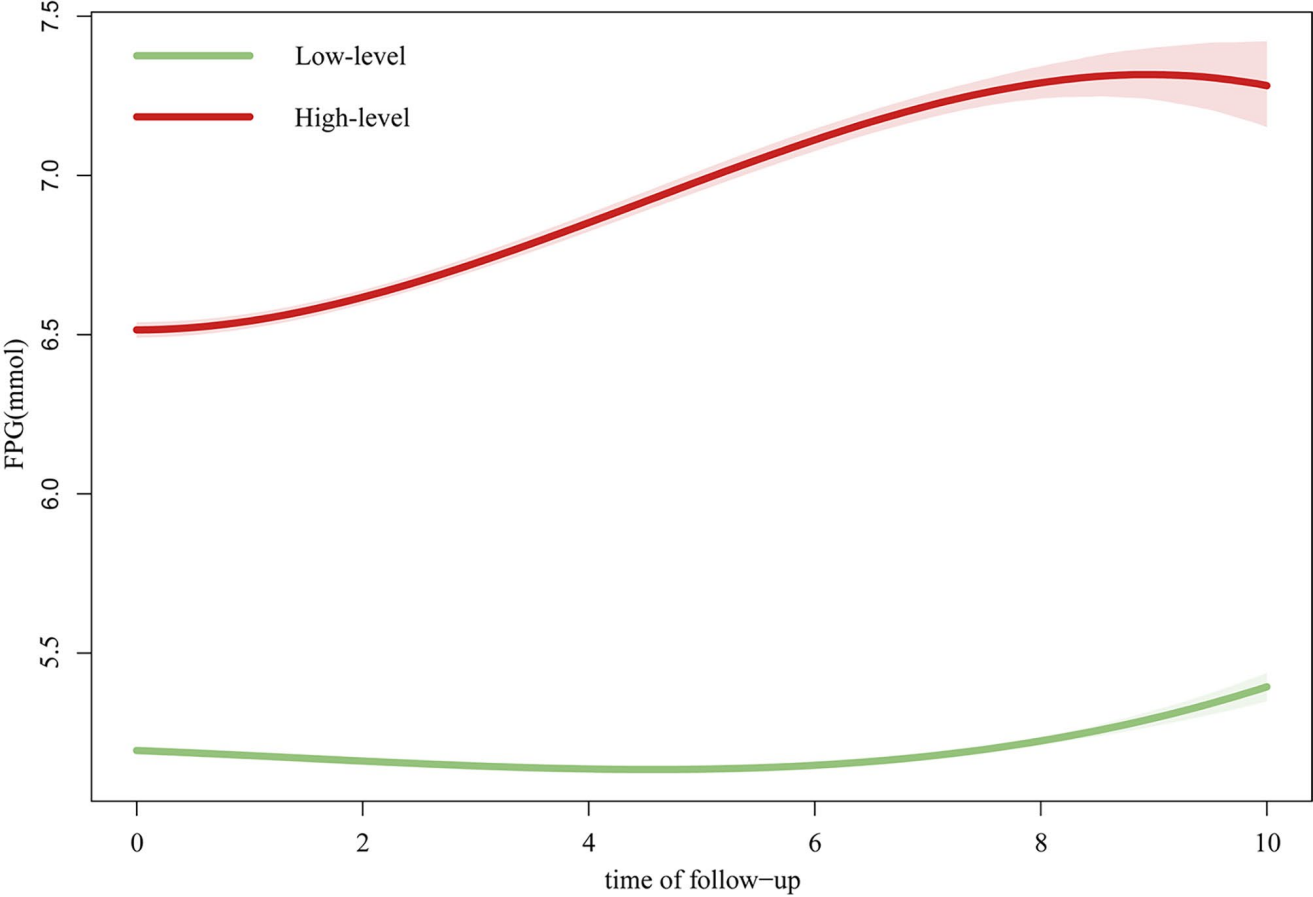
Trajectories of FPG over the follow-up time. The green shaded area represents the 95% confidence interval for the FPG. The latent class growth mixture modeling (LCGMM) was used to explore heterogeneity in the dynamic course of FPG to distinguish subgroups of similar underlying FPG trajectories as experienced over time. Abbreviation: FPG, fasting plasma glucose

Based on the BIC, class membership posterior probabilities, and classification to assess the goodness-of-fit of the competing LCGMM models (Table S2), the model with two FPG trajectories among the 51,479 participants was identified as the best-fit model: there were low-level (mean FPG = 5.12mmol/L, *n* = 39,291), and high-level (mean FPG = 6.74mmol/L, *n* = 12,188) trajectories (Fig. 1). High-level class had a lower proportion of participants (> 20%), which had highly discriminated with high mean posterior probabilities and posterior probabilities (> 90%). Compared with participants in the low-level class, counterparts in another group were more likely to be men with higher FPG, BMI, and waist circumference values (Table S3).

**Fig. 2:**
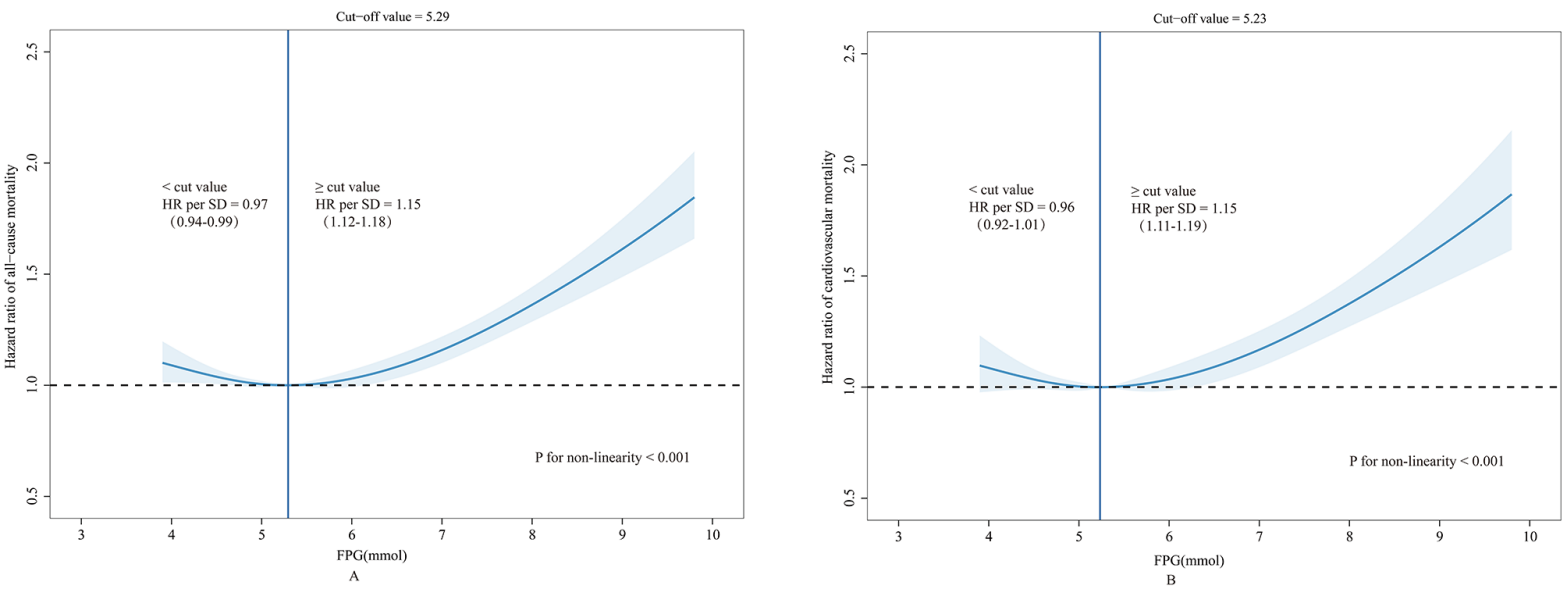
Restricted cubic spline plots of the relationship between FPG at baseline of all-cause mortality **(A)** and cardiovascular mortality **(B)**. The curve was computed using restricted cubic spline (RCS) function that took into account variables including sex, age, marital status, BMI, smoking status, alcohol drinking level, physical activity and hypertension. The green shaded area represents the 95% confidence interval for the HR. The dotted line shows the level at which the HR value is equal to 1

First, a U-shaped trend in the association between FPG at baseline and all-cause and cardiovascular mortality was observed in the study, and the dose-response relationships modeled by restricted cubic spline models in the middle-aged and elderly population were presented in Figs. 2, A and B. The cut-off values of all-cause and cardiovascular mortality were 5.29 and 5.23, respectively, and while FPG < 5.23mmol/L, the HR for cardiovascular mortality was 0.96(0.92–1.01) as per 1 SD FPG higher, *P* < 0.001. As FPG was more than 5.23mmol/L, the HR for cardiovascular mortality was 1.15(1.11–1.19) as per 1 SD FPG higher, *P* < 0.001. Similar results were observed for cardiovascular all-cause mortality, and while FPG < 5.29mmol/L, the HR for all-cause mortality was 0.97(0.94–0.99) as per 1 SD FPG higher, *P* < 0.001. As FPG was more than 5.29mmol/L, the HR for all-cause mortality was 1.15(1.12–1.18) as per 1 SD FPG higher, *P* < 0.001. Furthermore, the estimated risk for all-cause and cardiovascular mortality by longitudinal trajectories of FPG are presented in Table 2. After being adjusted for potential confounders, compared with the low-level category, the HRs for all-cause and cardiovascular mortality were 1.23(1.16–1.30) and 1.25(1.16–1.35), respectively, for the high-level group. For the analysis of the risk of cardiovascular mortality, compared with the low-level category, the HRs for CHD and stroke mortality were 1.19(1.08,1.30) and 1.33(1.18,1.51), respectively, for the high-level group. As seen in Figures S1 and S2, model three had excellent discriminative power over the follow-up period after being adjusted for potential confounders.

**Table 2:** Cox regression analysis between trajectories of FPG and all-cause mortality and cardiovascular mortality

Outcomes	Variables	No. of deaths	No. of person-years	Cumulative mortality rate^Т^	HRs (95% CIs)		
					Model 1	Model 2	Model 3
All-cause mortality	Low-level	6761	246955.4	27.4	1.00 (ref)	1.00 (ref)	1.00 (ref)
	High-level	2306	75262.6	30.6	1.27(1.21,1.33)	1.29(1.23,1.36)	1.23(1.16,1.30)
CVD mortality	Low-level	3536	246955.4	14.32	1.00 (ref)	1.00 (ref)	1.00 (ref)
	High-level	1276	75262.6	16.95	1.34(1.25,1.43)	1.32(1.24,1.41)	1.25(1.16,1.35)
CHD mortality	Low-level	2527	246955.4	10.23	1.00 (ref)	1.00 (ref)	1.00 (ref)
	High-level	868	75262.6	11.53	1.28(1.19,1.39)	1.29(1.19,1.39)	1.19(1.08,1.30)
Stroke mortality	Low-level	1207	246955.4	4.89	1.00 (ref)	1.00 (ref)	1.00 (ref)
	High-level	471	75262.6	6.26	1.42(1.27,1.58)	1.34(1.21,1.50)	1.33(1.18,1.51)

^Т^Per 1000 person-years. Model 1: Adjusted for age and gender. Model 2: Adjusted for age, gender, marital status, BMI, smoking, alcohol consumption, physical activity and hypertension. Model 3: Adjusted for age, gender, marital status, BMI, smoking, alcohol consumption, physical activity, hypertension and FPG.

The results of the subgroup analyses according to sex is presented in Tables S4, S5, S6 and S7. First, the gender subgroup analysis revealed that the cumulative mortality rates were higher in the male participants compared to the female counterparts. Also, after being adjusted for potential confounders, compared with the low-level FPG trajectory category of the respective group, the HRs for all-cause and cardiovascular mortality were higher in the male group. Furthermore, the sensitivity analyses showed similar results to the primary analysis, which are presented in Table S8.

## Discussion

The study had a median follow-up of 6.26 years (range 5.16–7.91), and all participants were examined at least four times. An approximately U-shaped trend in the association between FPG and all-cause and cardiovascular mortality was observed. The all-cause and cardiovascular mortality were lowest when the FPG was 5.29 and 5.23mmol/L, respectively. During the follow-up period, according to the FPG trajectory of the study participants, we divided them into two groups: low-level and high-level. As seen from the trajectory curves, the low-level group decreased first and then increased over the follow-up time, while the high-level group showed a smooth increasing trend.

Many previous studies have revealed the association between high FPG and cardiovascular mortality risk, with one noting that high FPG was the third leading risk factor for all-cause mortality from 1990 to 2017 [11, 26]. Multiple mechanisms of action between abnormal glucose metabolism and CVD/cardiometabolic risk have been suggested. First, abnormal glucose metabolism can disrupt normal endothelial function, accelerate atherosclerotic plaque formation, and contribute to plaque rupture and subsequent thrombosis, thereby increasing the risk of macrovascular mortality [11, 27]. Second, abnormal glucose metabolism may increase the risk of microvascular complications, cancer, heart failure, myocardial infarction and stroke [11, 27–29]. Third, increased blood glucose may lead to infarct expansion by several maladaptive metabolic pathways and an increased all-cause mortality [26, 27, 30, 31]. Although high FPG has been proven to be related to multiple noncommunicable diseases, including type 2 diabetes, coronary heart disease, and stroke, the association between FPG and all-cause mortality remains controversial: a J- or U-shaped relationship was recently reported [6, 32]. This suggests that the method of classifying FPG into predefined categories for study based on established criteria or quartiles may be flawed [33, 34]. It has been reported that this classification method may lead to the misclassification of those individuals close to the classification cutoff point [35, 36]. Whereas in the LGCMM model in this study, it was assumed that there was no single developmental curve in the study population and that individuals belonged to different subgroups with different developmental trajectories. The pattern of FPG changes during the follow-up period was modeled, based on the population heterogeneity.

In recent years, many studies on the trajectory analysis of FPG and CVD have been reported. For instance, Yuan Zhongxiang et al. identified three different trajectories of FPG and found that distinct trajectories of long-term normal FPG are associated with the development of CVD [37]. This suggests that the long-term pattern of FPG may potentially influence cardiovascular risk and is consistent with the“ticking clock” hypothesis proposed by Steven M et al. [38]. More recently, Dankang Li et al. identified five different trajectories of FPG and found that individuals with elevated-level trajectory patterns had a higher lifetime risk of CVD [39]. A subgroup analysis based on genders conducted by Ogata et al. revealed that similar FPG trajectory patterns were found in both sexes. Moreover, higher FPG levels were associated with an increased risk of CVD over time, especially in men, and this is consistent with our study [7]. Our study found that over 75% of the participants in the high-level group were in a state of impaired fasting glucose (IFG), which has also been described as a simply “prediabetes“ [9, 40]. By contrast, only about 20% of the study subjects in the low-level group were in a state of IFG. Many studies have suggested a higher risk of cardiovascular and all-cause mortality in the prediabetic population, which is also consistent with our findings [9, 27]. From the perspective of community-based primary health care and primary prevention of CVD, the primary aim is to target two risk factors, obesity and physical inactivity, after identifying high-risk groups [40, 41]. Studies in Asian populations have found that lifestyle interventions can significantly reduce cardiovascular risk and facilitate self-management of health [42, 43].

Our study has important implications for the primary prevention of cardiovascular mortality in the middle-aged and elderly and for public health. First, because of the U-shaped trend in the association between FPG and cardiovascular mortality, from an individual level, maintaining the FPG at around 5.23 for a long period may have the lowest risk. Second, this research reveals long-term patterns of FPG that may potentially impact all-cause and cardiovascular mortality. Considering that CVD prevention is long-term and dynamic, especially with increasing age and accumulation of co-morbidities, our results emphasize that dynamic surveillance and multi-level prevention should be implemented on a long-term or even lifetime basis. Furthermore, a longitudinal trajectory study with repeated measures and long-term follow-up should be a component of a distinct approach to identifying people at high risk for CVD. With the increased emphasis on primary health care and accelerating global aging trends, the FPG trajectory may be incorporated into primary health care as a new risk factor, and future research on its relationship with other health conditions should be strengthened.

Strengths of this study include the cohort design, the repeated measurements of FPG, the robustness of the observed associations, and the identification of groups of individuals with similar patterns of FPG trajectories based on long-term follow-up and repeated measurements. On the other hand, several limitations of the study are worth mentioning. First, the study was conducted among middle-aged and elderly Chinese individuals with an average age of approximately 65.4 years, making it difficult to generalize to all populations. Second, although we have adjusted for some confounders as far as possible, the possibility of bias still exists, such as the use of antidiabetic, antihypertension drugs and other medications, dietary factors, genetic factors, and unavoidable recall bias. In fact, in most developing countries, data on 2-hour PG and hemoglobin A1c are often difficult to obtain from large routine health checks because they are expensive and inconvenient. Therefore, the association between the longitudinal trajectories of these two indicators and CVD and other disease conditions needs to be further studied.

## Conclusions

Overall, as an important indicator of the overall glycemic state, a U-shaped association between FPG and all-cause and cardiovascular mortality was observed in the study. In addition, the long-term trajectory study found that higher FPG levels are associated with an increased risk of all-cause and cardiovascular mortality over time, while there may be some potential effects of long-term patterns of FPG. As an indicator preceding the onset of metabolic diseases, the prognostic capacity of FPG for CVD risk can be a complementary tool for public health primary prevention, but more studies are still needed.

### Electronic supplementary material

Below is the link to the electronic supplementary material.

Supplementary Material 1. **Supplementary Materials**: Table S1: The records and times of the four examinations; Table S2: Latent Class Growth Mixture models (LCGMM) results; Table S3: Baseline characteristics of participants according to the trajectories of FPG; Table S4: Latent Class Growth Mixture models (LCGMM) results of men; S5: The results of the subgroup analyses according to sex in men; Table S6: Latent Class Growth Mixture models (LCGMM) results of women; Table S7: The results of the subgroup analyses according to sex in women; Table S8: Latent Class Growth Mixture models (LCGMM) results; Table S9 Cox regression analysis between trajectories of FPG and all-cause mortality and cardiovascular mortality after excluding those participants with less than 4 years of follow-up

## Data Availability

No datasets were generated or analysed during the current study.

## References

[1] Roth GA, Forouzanfar MH, Moran AE, Barber R, Nguyen G, Feigin VL (2015). Demographic and epidemiologic drivers of global cardiovascular mortality. N Engl J Med.

[2] Ren J, Zhang Y (2018). Targeting Autophagy in Aging and Aging-Related Cardiovascular diseases. Trends Pharmacol Sci.

[3] Shaw LJ, Goyal A, Mehta C, Xie J, Phillips L, Kelkar A (2018). 10-Year resource utilization and costs for Cardiovascular Care. J Am Coll Cardiol.

[4] Liu S, Li Y, Zeng X, Wang H, Yin P, Wang L (2019). Burden of Cardiovascular diseases in China, 1990–2016: findings from the 2016 global burden of Disease Study. JAMA Cardiol.

[5] Park C, Guallar E, Linton JA, Lee DC, Jang Y, Son DK (2013). Fasting glucose level and the risk of incident atherosclerotic cardiovascular diseases. Diabetes Care.

[6] Mongraw-Chaffin M, LaCroix AZ, Sears DD, Garcia L, Phillips LS, Salmoirago-Blotcher E (2017). A prospective study of low fasting glucose with cardiovascular disease events and all-cause mortality: the women’s Health Initiative. Metabolism.

[7] Ogata S, Watanabe M, Kokubo Y, Higashiyama A, Nakao YM, Takegami M (2019). Longitudinal trajectories of fasting plasma glucose and risks of Cardiovascular diseases in Middle Age to Elderly people within the General Japanese Population: the Suita Study. J Am Heart Assoc.

[8] Kayama Y, Raaz U, Jagger A, Adam M, Schellinger IN, Sakamoto M (2015). Diabetic Cardiovascular Disease Induced by oxidative stress. Int J Mol Sci.

[9] Tabak AG, Herder C, Rathmann W, Brunner EJ, Kivimaki M (2012). Prediabetes: a high-risk state for diabetes development. Lancet.

[10] Turin TC, Okamura T, Rumana N, Afzal AR, Watanabe M, Higashiyama A (2017). Diabetes and lifetime risk of stroke and subtypes in an urban middle-aged population. J Diabetes Complications.

[11] Liu L, Chen X, Liu Y, Sun X, Yin Z, Li H (2019). The association between fasting plasma glucose and all-cause and cause-specific mortality by gender: the rural Chinese cohort study. Diabetes Metab Res Rev.

[12] Lu J, He J, Li M, Tang X, Hu R, Shi L (2019). Predictive value of fasting glucose, Postload Glucose, and Hemoglobin A(1c) on risk of diabetes and complications in Chinese adults. Diabetes Care.

[13] Becque MD, Katch VL, Rocchini AP, Marks CR, Moorehead C (1988). Coronary risk incidence of obese adolescents: reduction by exercise plus diet intervention. Pediatrics.

[14] Zheng Y, Song M, Manson JE, Giovannucci EL, Hu FB (2017). Group-based trajectory of body shape from ages 5 to 55 years and Cardiometabolic Disease Risk in 2 US cohorts. Am J Epidemiol.

[15] Nagin DS (2014). Group-based trajectory modeling: an overview. Ann Nutr Metab.

[16] Buscot MJ, Thomson RJ, Juonala M, Sabin MA, Burgner DP, Lehtimaki T (2018). Distinct child-to-adult body mass index trajectories are associated with different levels of adult cardiometabolic risk. Eur Heart J.

[17] Lee J, Song RJ, Musa Yola I, Shrout TA, Mitchell GF, Vasan RS (2021). Association of Estimated Cardiorespiratory Fitness in midlife with cardiometabolic outcomes and mortality. JAMA Netw Open.

[18] Jin C, Chen S, Vaidya A, Wu Y, Wu Z, Hu FB (2017). Longitudinal change in fasting blood glucose and myocardial infarction risk in a Population without Diabetes. Diabetes Care.

[19] Lee CL, Sheu WH, Lee IT, Lin SY, Liang WM, Wang JS (2018). Trajectories of fasting plasma glucose variability and mortality in type 2 diabetes. Diabetes Metab.

[20] Bai K, Chen X, Shi Z, He K, Hu X, Song R (2022). Hypertension modifies the associations of body mass index and waist circumference with all-cause mortality among older Chinese: a retrospective cohort study. BMC Geriatr.

[21] He K, Zhang W, Hu X, Zhao H, Guo B, Shi Z (2021). Relationship between multimorbidity, disease cluster and all-cause mortality among older adults: a retrospective cohort analysis. BMC Public Health.

[22] Song R, Chen X, He K, Hu X, Bai K, Shi W (2022). Associations of BMI with all-cause mortality in normoglycemia, impaired fasting glucose and type 2 diabetes mellitus among an elderly Chinese population: a cohort study. BMC Geriatr.

[23] He K, Zhang W, Hu X, Zhao H, Song R, Bai K (2022). Stronger associations of Body Mass Index and Waist circumference with diabetes than Waist-Height ratio and triglyceride glucose index in the Middle-aged and Elderly Population: a retrospective cohort study. J Diabetes Res.

[24] Wu M, Yu X, Xu L, Wu S, Tian Y (2022). Associations of longitudinal trajectories in body roundness index with mortality and cardiovascular outcomes: a cohort study. Am J Clin Nutr.

[25] Marioni RE, Proust-Lima C, Amieva H, Brayne C, Matthews FE, Dartigues JF (2014). Cognitive lifestyle jointly predicts longitudinal cognitive decline and mortality risk. Eur J Epidemiol.

[26] Collaborators GBDRF. Global, regional, and national comparative risk assessment of 84 behaviournvironmental and occupational, and metabolic risks or clusters of risks for 195 countries and territories, 1990–2017: a systematic analysis for the Global Burden of Disease Study 2017. Lancet. 2018;392(10159):1923-94. Epub 20181108. 10.1016/S0140-6736(18)32225-6. 10.1016/S0140-6736(18)32225-6 PMC6227755 30496105

[27] Nakagami T, Group DS (2004). Hyperglycaemia and mortality from all causes and from cardiovascular disease in five populations of Asian origin. Diabetologia.

[28] Lehto S, Ronnemaa T, Pyorala K, Laakso M (1996). Predictors of stroke in middle-aged patients with non-insulin-dependent diabetes. Stroke.

[29] Turner RC, Millns H, Neil HA, Stratton IM, Manley SE, Matthews DR (1998). Risk factors for coronary artery disease in non-insulin dependent diabetes mellitus: United Kingdom prospective diabetes study (UKPDS: 23). BMJ.

[30] Gilmore RM, Stead LG (2006). The role of hyperglycemia in acute ischemic stroke. Neurocrit Care.

[31] Stratton IM, Adler AI, Neil HA, Matthews DR, Manley SE, Cull CA (2000). Association of glycaemia with macrovascular and microvascular complications of type 2 diabetes (UKPDS 35): prospective observational study. BMJ.

[32] Emerging Risk Factors Collaboration S, Gao N, Seshasai P, Gobin SR, Kaptoge R, Di Angelantonio S, Ingelsson E, Lawlor E, Selvin DA, Stampfer E, Stehouwer M, Lewington CD, Pennells S, Thompson L, Sattar A, White N, Ray IR, K. K., Danesh J. Diabetes mellitus, fasting blood glucose concentration, and risk of vascular disease: a collaborative meta-analysis of 102 prospective studies. Lancet (London, England),. (2010).375(9733),:2215–22. 10.1016/S0140-6736(10)60484-9. 10.1016/S0140-6736(10)60484-9 PMC2904878 20609967

[33] Preiss D, Welsh P, Murray HM, Shepherd J, Packard C, Macfarlane P (2010). Fasting plasma glucose in non-diabetic participants and the risk for incident cardiovascular events, diabetes, and mortality: results from WOSCOPS 15-year follow-up. Eur Heart J.

[34] Sinha A, Ning H, Ahmad FS, Bancks MP, Carnethon MR, O’Brien MJ (2021). Association of fasting glucose with lifetime risk of incident heart failure: the Lifetime Risk Pooling Project. Cardiovasc Diabetol.

[35] Dhana K, van Rosmalen J, Vistisen D, Ikram MA, Hofman A, Franco OH (2016). Trajectories of body mass index before the diagnosis of cardiovascular disease: a latent class trajectory analysis. Eur J Epidemiol.

[36] Streiner DL (2002). Breaking up is hard to do: the heartbreak of dichotomizing continuous data. Can J Psychiatry.

[37] Yuan Z, Yang Y, Wang C, Liu J, Sun X, Liu Y, et al. Trajectories of long-term normal fasting plasma glucose and risk of Coronary Heart Disease: a prospective cohort study. J Am Heart Association. 2018;7(4). 10.1161/jaha.117.007607. 10.1161/JAHA.117.007607 PMC5850191 29440033

[38] Haffner SM, Stern MP, Hazuda HP, Mitchell BD, Patterson JK (1990). Cardiovascular risk factors in confirmed prediabetic individuals. Does the clock for coronary heart disease start ticking before the onset of clinical diabetes?. JAMA.

[39] Li D, Song L, Wang L, Chen S, Yang Y, Hu Y et al. Association of fasting plasma glucose trajectory with lifetime risk of cardiovascular disease. Eur J Clin Nutr. 2022. Epub 20221128. 10.1038/s41430-022-01243-x. 10.1038/s41430-022-01243-x 36443394

[40] American Diabetes A (2013). Diagnosis and classification of diabetes mellitus. Diabetes Care.

[41] DeFronzo RA, Abdul-Ghani MA (2011). Preservation of beta-cell function: the key to diabetes prevention. J Clin Endocrinol Metab.

[42] Ramachandran A, Snehalatha C, Mary S, Mukesh B, Bhaskar AD, Vijay V (2006). The Indian Diabetes Prevention Programme shows that lifestyle modification and metformin prevent type 2 diabetes in Asian Indian subjects with impaired glucose tolerance (IDPP-1). Diabetologia.

[43] Jenum AK, Brekke I, Mdala I, Muilwijk M, Ramachandran A, Kjollesdal M (2019). Effects of dietary and physical activity interventions on the risk of type 2 diabetes in South asians: meta-analysis of individual participant data from randomised controlled trials. Diabetologia.

