# Supplementary material for: Association of longitudinal trajectories of fasting plasma glucose with all-cause and cardiovascular mortality among a Chinese older population: a retrospective cohort study
Source: BMC Public Health. 2024 May 17;24:1335. doi: 10.1186/s12889-024-18823-0 (PMC11102116; doi:10.1186/s12889-024-18823-0)
Supplement: Supplementary file 1 — Supplementary Material 1. Supplementary Materials: Table S1: The records and times of the four examinations; Table S2: Latent Class Growth Mixture models (LCGMM) results; Table S3: Baseline characteristics of participants according to the trajectories of FPG; Table S4: Latent Class Growth Mixture models (LCGMM) results of men; S5: The results of the subgroup analyses according to sex in men; Table S6: Latent Class Growth Mixture models (LCGMM) results of women; Table S7: The results of the subgroup analyses according to sex in women; Table S8: Latent Class Growth Mixture models (LCGMM) results; Table S9 Cox regression analysis between trajectories of FPG and all-cause mortality and cardiovascular mortality after excluding those participants with less than 4 years of follow-up [file 12889_2024_18823_MOESM1_ESM.docx]

Supplementary Material

**Association of longitudinal trajectories of Fasting Plasma Glucose with all-cause and cardiovascular mortality among a Chinese older population: a retrospective cohort study**

**Xuejiao Chen^1^, Jiacheng Ding^1^, Zhan Shi^2^, Kaizhi Bai^1^, Songhe Shi^1^,Qingfeng Tian^1^***

*** Correspondence:** Qingfeng Tian，zzutqf@126.com

**Table S1** The records and times of the examinations

|  | 2010 | 2011 | 2012 | 2013 | 2014 | 2015 | 2016 | 2017 | 2018 | 2019 |
| --- | --- | --- | --- | --- | --- | --- | --- | --- | --- | --- |
| 2010 | 7442 | 4757 | 5432 | 7072 | 5870 | 5682 | 4917 | 5262 | 4440 | 4808 |
| 2011 |  | 11554 | 6841 | 10642 | 8280 | 9539 | 8755 | 9013 | 8396 | 8779 |
| 2012 |  |  | 7214 | 6619 | 5779 | 6336 | 5726 | 5807 | 5355 | 5559 |
| 2013 |  |  |  | 17480 | 9110 | 14983 | 11755 | 15130 | 13992 | 14994 |
| 2014 |  |  |  |  | 1925 | 1728 | 1534 | 1704 | 1662 | 1670 |
| 2015 |  |  |  |  |  | 4839 | 2285 | 4675 | 4583 | 4666 |
| 2016 |  |  |  |  |  |  | 1025 | 1025 | 1025 | 1025 |

**Table S2** Latent Class Growth Mixture models (LCGMM) results

| Nb. Latent  classes | Polynomial  degree | Log-Lik | Entropy | BIC | % Participants per class | Mean posterior probabilities | Posterior probabilities>0.7 (%) |
| --- | --- | --- | --- | --- | --- | --- | --- |
| 1 | Linear | -474231.4 | 1.0000000 | 948549.5 | 100 | n.a | n.a |
|  | Quadratic | -473721.0 | 1.0000000 | 947583.1 | 100 | n.a | n.a |
|  | Cubic | -473476.7 | 1.0000000 | 947127.0 | 100 | n.a | n.a |
| 2 | Linear | -457757.9 | 0.6990330 | 915646.0 | 77.27/22.73 | 0.93/0.92 | 95.48/89.05 |
|  | Quadratic | -454347.5 | 0.7393502 | 908890.4 | 76.81/23.19 | 0.94/0.93 | 95.86/90.10 |
|  | **Cubic** | **-451456.0** | **0.7605412** | **903150.7** | **76.32/23.68** | **0.94/0.93** | **96.20/90.65** |
| 3 | Linear | -456621.6 | 0.6143262 | 913416.7 | 18.52/65.58/15.9 | 0.67/0.86/0.90 | 41.58/86.14/86.22 |
|  | Quadratic | -452361.0 | 0.6631844 | 904971.6 | 18.93/64/17.07 | 0.70/0.88/0.92 | 49.26/87.34/88.85 |
|  | Cubic | -448952.4 | 0.6816875 | 898208.6 | 19.31/62.75/17.93 | 0.72/0.88/0.93 | 55.16/87.54/89.55 |
| 4 | Linear | -456496.0 | 0.4735248 | 913209.1 | 13.56/24.63/48.06/13.75 | 0.61/0.53/0.71/0.88 | 26.37/1.49/52.95/81.62 |
|  | Quadratic | -452119.2 | 0.5386055 | 904542.2 | 9.73/26.97/47.74/15.56 | 0.61/0.59/0.76/0.89 | 26.20/14.58/64.92/83.21 |
|  | Cubic | -448986.0 | 0.5287812 | 898341.0 | 9.43/32.72/39.74/18.11 | 0.62/0.60/0.73/0.90 | 27.86/18.16/57.62/84.46 |

Reported are: the number of latent class considered, the polynomial form of the model, the maximum Log-Likelihood (Log-Lik), Entropy, the Bayesian information Criterion (BIC), and for models with 2 or more classes, the a-posteriori classification of subjects in each class (%), the mean of posterior probabilities in each latent class, and the % of subjects classified in each class with a posterior probability above 0.7. The best fitting model is highlighted in bold characters. (n.a: not applicable).

**Table S3** Baseline characteristics of participants according to the trajectories of FPG

| Characteristics | Low-level | High-level | P |
| --- | --- | --- | --- |
| Num | 39291 | 12188 |  |
| Age (years) | 65.68 (62.18, 72.17) | 64.63 (61.50, 70.30) | <0.001 |
| Gender (%) |  |  | <0.001 |
| Women | 20860 (53.09) | 6932 (56.88) |  |
| Men | 18431 (46.91) | 5256 (43.12) |  |
| Marital status (%) |  |  | <0.001 |
| Living without partner | 9725 (24.75) | 2604 (21.37) |  |
| Living with partner | 29566 (75.25) | 9584 (78.63) |  |
| Smoking (%) |  |  | 0.004 |
| Never or previous | 33403 (85.01) | 10491 (86.08) |  |
| Current | 5888 (14.99) | 1697 (13.92) |  |
| Drinking (%) |  |  | <0.001 |
| Never | 36593 (93.13) | 11181 (91.74) |  |
| Occasionally | 1871 (4.76) | 558 (4.58) |  |
| Daily | 827 (2.10) | 449 (3.68) |  |
| Physical activity (%) |  |  | <0.001 |
| Never | 29666 (75.50) | 8676 (71.18) |  |
| Occasionally | 3748 (9.54) | 1390 (11.40) |  |
| Daily | 5877 (14.96) | 2122 (17.41) |  |
| Hypertension |  |  | <0.001 |
| No | 19730 (50.22) | 4419 (36.26) |  |
| Yes | 19561 (49.78) | 7769 (63.74) |  |
| FPG | 5.17 (4.70, 5.60) | 6.32 (5.50, 7.60) | <0.001 |
| meanFPG | 5.12 (4.84, 5.43) | 6.74 (6.25, 7.49) | <0.001 |
| WC | 82.00 (76.00, 88.00) | 85.00 (79.20, 92.00) | <0.001 |
| BMI | 23.81 (22.04, 26.12) | 25.07 (23.11, 27.34) | <0.001 |
| Time of follow-up | 6.04 (5.34, 7.92) | 6.05 (5.00, 7.81) | <0.001 |

Abbreviations: FPG, fasting plasma glucose; BMI, body mass index; WC, waist circumference

Data are presented as median (interquartile range), or number (percentage).

**Table S4** Latent Class Growth Mixture models (LCGMM) results of men

| Nb. Latent  classes | Polynomial  degree | Log-Lik | Entropy | BIC | % Participants per class | Mean posterior probabilities | Posterior probabilities>0.7 (%) |
| --- | --- | --- | --- | --- | --- | --- | --- |
| 1 | Linear | -213171.5 | 1.0000000 | 426403.4 | 100 | n.a | n.a |
|  | Quadratic | -212915.2 | 1.0000000 | 425931.1 | 100 | n.a | n.a |
|  | Cubic | -212773.3 | 1.0000000 | 425697.6 | 100 | n.a | n.a |
| 2 | Linear | -205129.2 | 0.7150988 | 410359.1 | 79.23/20.77 | 0.93/0.67 | 96.04/88.84 |
|  | Quadratic | -203413.3 | 0.7544260 | 406977.7 | 78.78/21.22 | 0.94/0.93 | 96.47/90.01 |
|  | **Cubic** | **-202124.4** | **0.7712057** | **404460.3** | **78.29/21.71** | **0.95/0.93** | **96.76/90.71** |
| 3 | Linear | -204620.8 | 0.6027002 | 409382.6 | 18.96/65.68/15.36 | 0.68/0.85/0.91 | 42.06/84.63/87.11 |
|  | Quadratic | -202510.4 | 0.6553783 | 405222.4 | 19.68/64.02/16.30 | 0.71/0.87/0.93 | 51.88/86.34/89.36 |
|  | Cubic | -200970.1 | 0.6817710 | 402212.1 | 19.97/62.98/17.06 | 0.73/0.88/0.93 | 57.57/87.14/89.75 |
| 4 | Linear | -204566.6 | 0.4612448 | 409314.5 | 13.35/33.09/40.64/12.92 | 0.63/0.55/0.69/0.89 | 31.28/3.87/45.78/83.20 |
|  | Quadratic | -202354.5 | 0.5975259 | 404960.7 | 8.04/20.32/58.45/13.18 | 0.62/0.60/0.82/0.88 | 28.20/19.30/77.65/82.52 |
|  | Cubic | -200866.6 | 0.5591438 | 402065.6 | 8.33/31.12/44.40/16.15 | 0.63/0.62/0.77/0.90 | 28.84/24.94/66.15/84.52 |

**Table S**5 The results of the subgroup analyses according to sex in men

| Outcomes | Variables | No. of deaths | No. of person-years | Cumulative  mortality rate^Т^ | HRs (95% CIs) | | |
| --- | --- | --- | --- | --- | --- | --- | --- |
|  |  |  |  |  | Model 1 | Model 2 | Model 3 |
| All-cause mortality | Low-level | 3715 | 115928.1 | 32.05 | 1.00 (ref) | 1.00 (ref) | 1.00 (ref) |
|  | High-level | 1117 | 31403.9 | 35.57 | 1.21(1.13,1.30) | 1.24(1.15,1.32) | 1.19(1.10,1.29) |
| Cardiovascular disease  mortality | Low-level | 1870 | 115928.1 | 16.13 | 1.00 (ref) | 1.00 (ref) | 1.00 (ref) |
|  | High-level | 610 | 31403.9 | 19.42 | 1.31(1.20,1.44) | 1.30(1.18,1.42) | 1.26(1.13,1.40) |
| Coronary heart disease  mortality | Low-level | 1323 | 115928.1 | 11.41 | 1.00 (ref) | 1.00 (ref) | 1.00 (ref) |
|  | High-level | 401 | 31403.9 | 12.77 | 1.23(1.10,1.37) | 1.24(1.11,1.39) | 1.19(1.04,1.36) |
| Stroke mortality | Low-level | 651 | 115928.1 | 5.62 | 1.00 (ref) | 1.00 (ref) | 1.00 (ref) |
|  | High-level | 239 | 31403.9 | 7.61 | 1.46(1.26,1.70) | 1.37(1.17,1.59) | 1.35(1.13,1.61) |

^Т^Per 1000 person-years. Model 1: Adjusted for age and gender. Model 2: Adjusted for age, gender, marital status, body mass index, smoking, alcohol consumption, physical activity and Hypertension. Model 3: Adjusted for age, gender, marital status, body mass index, smoking, alcohol consumption, physical activity. Hypertension and FPG.

**Table S6** Latent Class Growth Mixture models (LCGMM) results of women

| Nb. Latent  classes | Polynomial  degree | Log-Lik | Entropy | BIC | % Participants per class | Mean posterior probabilities | Posterior probabilities>0.7 (%) |
| --- | --- | --- | --- | --- | --- | --- | --- |
| 1 | Linear | -213171.5 | 1.0000000 | 426403.4 | 100 | n.a | n.a |
|  | Quadratic | -260672.5 | 1.0000000 | 521447.2 | 100 | n.a | n.a |
|  | Cubic | -212773.3 | 1.0000000 | 425697.6 | 100 | n.a | n.a |
| 2 | Linear | -205129.2 | 0.7150988 | 410359.1 | 79.23/20.77 | 0.93/0.92 | 96.04/88.84 |
|  | Quadratic | -250754.6 | 0.7281392 | 501662.6 | 75.06/24.94 | 0.93/0.93 | 95.46/90.04 |
|  | **Cubic** | **-202124.4** | **0.7712057** | **404460.3** | **78.29/21.71** | **0.95/0.05** | **96.76/90.71** |
| 3 | Linear | -204620.8 | 0.6027002 | 409382.6 | 18.96/65.68/15.36 | 0.68/0.85/0.91 | 42.06/84.63/87.11 |
|  | Quadratic | -249682.5 | 0.6669310 | 499569.7 | 18.60/63.64/17.76 | 0.70/0.88/0.92 | 48.59/87.94/87.93 |
|  | Cubic | -200970.1 | 0.6817710 | 402212.1 | 19.97/62.98/17.06 | 0.73/0.88/0.93 | 57.57/87.14/89.75 |
| 4 | Linear | -204566.6 | 0.4612448 | 409314.5 | 13.35/33.09/40.64/12.92 | 0.63/0.55/0.69/0.89 | 31.28/3.87/45.78/83.20 |
|  | Quadratic | -249593.8 | 0.7194788 | 499443.5 | 18.94/17.16/63.32/0.58 | 0.70/0.89/0.88/0.72 | 48.10/85.13/87.55/48.45 |
|  | Cubic | -200866.6 | 0.5591438 | 402065.6 | 8.33/31.12/44.40/16.15 | 0.63/0.62/0.77/0.90 | 28.84/24.94/66.15/84.52 |

Reported are: the number of latent class considered, the polynomial form of the model, the maximum Log-Likelihood (Log-Lik), Entropy, the Bayesian information Criterion (BIC), and for models with 2 or more classes, the a-posteriori classification of subjects in each class (%), the mean of posterior probabilities in each latent class, and the % of subjects classified in each class with a posterior probability above 0.7. The best fitting model is highlighted in bold characters. (n.a: not applicable).

**Table S7** The results of the subgroup analyses according to sex in women

| Outcomes | Variables | No. of deaths | No. of person-years | Cumulative  mortality rate^Т^ | HRs (95% CIs) | | |
| --- | --- | --- | --- | --- | --- | --- | --- |
|  |  |  |  |  | Model 1 | Model 2 | Model 3 |
| All-cause mortality | Low-level | 3047 | 130884 | 23.28 | 1.00 (ref) | 1.00 (ref) | 1.00 (ref) |
|  | High-level | 1188 | 44002.03 | 27.00 | 1.34(1.25,1.43) | 1.37(1.28,1.46) | 1.27(1.17,1.38) |
| Cardiovascular disease mortality | Low-level | 1666 | 130884 | 12.73 | 1.00 (ref) | 1.00 (ref) | 1.00 (ref) |
|  | High-level | 666 | 44002.03 | 15.15 | 1.36(1.25,1.50) | 1.36(1.24,1.49) | 1.25(1.12,1.39) |
| Coronary heart disease mortality | Low-level | 1204 | 130884 | 9.20 | 1.00 (ref) | 1.00 (ref) | 1.00 (ref) |
|  | High-level | 467 | 44002.03 | 10.61 | 1.35(1.21,1.50) | 1.35(1.21,1.51) | 1.20(1.05,1.36) |
| Stroke mortality | Low-level | 558 | 130884 | 4.26 | 1.00 (ref) | 1.00 (ref) | 1.00 (ref) |
|  | High-level | 230 | 44002.03 | 5.27 | 1.36(1.17,1.59) | 1.31(1.12,1.53) | 1.30(1.08,1.56) |

^Т^Per 1000 person-years. Model 1: Adjusted for age and gender. Model 2: Adjusted for age, gender, marital status, body mass index, smoking, alcohol consumption, physical activity and Hypertension. Model 3: Adjusted for age, gender, marital status, body mass index, smoking, alcohol consumption, physical activity. Hypertension and FPG.

**Table S8** Latent Class Growth Mixture models (LCGMM) results

| Nb. Latent  classes | Polynomial  degree | Log-Lik | | Entropy | | BIC | | % Participants per class | | Mean posterior probabilities | | Posterior probabilities>0.7 (%) | |
| --- | --- | --- | --- | --- | --- | --- | --- | --- | --- | --- | --- | --- | --- |
| 1 | Linear | | -425102.4 | | 1.0000000 | | 850290.3 | | 100 | | n.a | | n.a |
|  | Quadratic | | -424620.0 | | 1.0000000 | | 849379.0 | | 100 | | n.a | | n.a |
|  | Cubic | | -424351.3 | | 1.0000000 | | 848873.7 | | 100 | | n.a | | n.a |
| 2 | Linear | | -410536.1 | | 0.7049196 | | 821200.5 | | 77.36/22.64 | | 0.93/0.92 | | 95.63/89.05 |
|  | Quadratic | | -407350.7 | | 0.7484020 | | 814893.8 | | 77.04/22.96 | | 0.94/0.93 | | 95.99/90.51 |
|  | **Cubic** | | -404749.9 | | **0.7687907** | | **809735.0** | | **76.60/23.40** | | **0.95/0.94** | | **96.19/91.03** |
| 3 | Linear | | -409552.1 | | 0.6182716 | | 819275.4 | | 18.53/65.43/16.05 | | 0.68/0.86/0.91 | | 43.40/86.24/86.27 |
|  | Quadratic | | -405562.5 | | 0.6717530 | | 811370.9 | | 19.08/64.00/16.92 | | 0.71/0.88/0.92 | | 53.92/87.76/89.18 |
|  | Cubic | | -402590.2 | | 0.6879448 | | 805479.9 | | 18.68/63.18/18.14 | | 0.72/0.89/0.93 | | 56.08/88.18/89.37 |
| 4 | Linear | | -409506.1 | | 0.4479832 | | 819226.0 | | 15.01/50.97/18.82/15.2 | | 0.63/0.59/0.61/0.89 | | 30.56/5.26/18.74/84.76 |
|  | Quadratic | | -405398.4 | | 0.5286742 | | 811096.2 | | 10.30/29.04/44.68/15.98 | | 0.62/0.59/0.75/0.89 | | 27.72/12.17/61.23/84.05 |
|  | Cubic | | -402433.3 | | 0.5685080 | | 805230.2 | | 8.38/27.03/47.81/16.76 | | 0.61/0.62/0.78/0.89 | | 25.14/22.69/69.92/83.32 |

Reported are: the number of latent class considered, the polynomial form of the model, the maximum Log-Likelihood (Log-Lik), Entropy, the Bayesian information Criterion (BIC), and for models with 2 or more classes, the a-posteriori classification of subjects in each class (%), the mean of posterior probabilities in each latent class, and the % of subjects classified in each class with a posterior probability above 0.7. The best fitting model is highlighted in bold characters. (n.a: not applicable).

**Table S9** Cox regression analysis between trajectories of FPG and all-cause mortality and cardiovascular mortality after excluding those participants with less than 4 years of follow-up

| Outcomes | Variables | No. of deaths | No. of person-years | Cumulative  mortality rate^Т^ | HRs (95% CIs) | | |
| --- | --- | --- | --- | --- | --- | --- | --- |
|  |  |  |  |  | Model 1 | Model 2 | Model 3 |
| All-cause mortality | Low-level | 5244 | 227113.7 | 23.09 | 1.00 (ref) | 1.00 (ref) | 1.00 (ref) |
|  | High-level | 1917 | 69096.2 | 27.74 | 1.33(1.26,1.40) | 1.35(1.28,1.43) | 1.29(1.21,1.37) |
| Cardiovascular disease  mortality | Low-level | 2816 | 227113.7 | 12.40 | 1.00 (ref) | 1.00 (ref) | 1.00 (ref) |
|  | High-level | 1085 | 69096.2 | 15.70 | 1.40(1.30,1.50) | 1.38(1.28,1.48) | 1.30(1.20,1.41) |
| Coronary heart disease  mortality | Low-level | 2064 | 227113.7 | 9.09 | 1.00 (ref) | 1.00 (ref) | 1.00 (ref) |
|  | High-level | 737 | 69096.2 | 10.67 | 1.30(1.20,1.42) | 1.31(1.20,1.43) | 1.20(1.09,1.33) |
| Stroke mortality | Low-level | 926 | 227113.7 | 4.08 | 1.00 (ref) | 1.00 (ref) | 1.00 (ref) |
|  | High-level | 404 | 69096.2 | 5.85 | 1.55(1.38, 1.74) | 1.47(1.30,1.66) | 1.44(1.26,1.66) |

^Т^Per 1000 person-years. Model 1: Adjusted for age and gender. Model 2: Adjusted for age, gender, marital status, body mass index, smoking, alcohol consumption, physical activity and Hypertension. Model 3: Adjusted for age, gender, marital status, body mass index, smoking, alcohol consumption, physical activity. Hypertension and FPG.
